# A prospective study of shoulder pain in primary care: Prevalence of imaged pathology and response to guided diagnostic blocks

**DOI:** 10.1186/1471-2474-12-119

**Published:** 2011-05-28

**Authors:** Angela Cadogan, Mark Laslett, Wayne A Hing, Peter J McNair, Mark H Coates

**Affiliations:** 1 Health & Rehabilitation Research Institute, AUT University, Akoranga Drive, Private Bag 92006, Northcote, Auckland, New Zealand; 2 Physiosouth, Christchurch, New Zealand; 3 Christchurch Radiology Group, Christchurch, New Zealand

## Abstract

**Background:**

The prevalence of imaged pathology in primary care has received little attention and the relevance of identified pathology to symptoms remains unclear. This paper reports the prevalence of imaged pathology and the association between pathology and response to diagnostic blocks into the subacromial bursa (SAB), acromioclavicular joint (ACJ) and glenohumeral joint (GHJ).

**Methods:**

Consecutive patients with shoulder pain recruited from primary care underwent standardised x-ray, diagnostic ultrasound scan and diagnostic injections of local anaesthetic into the SAB and ACJ. Subjects who reported less than 80% reduction in pain following either of these injections were referred for a magnetic resonance arthrogram (MRA) and GHJ diagnostic block. Differences in proportions of positive and negative imaging findings in the anaesthetic response groups were assessed using Fishers test and odds ratios were calculated a for positive anaesthetic response (PAR) to diagnostic blocks.

**Results:**

In the 208 subjects recruited, the rotator cuff and SAB displayed the highest prevalence of pathology on both ultrasound (50% and 31% respectively) and MRA (65% and 76% respectively). The prevalence of PAR following SAB injection was 34% and ACJ injection 14%. Of the 59% reporting a negative anaesthetic response (NAR) for both of these injections, 16% demonstrated a PAR to GHJ injection. A full thickness tear of supraspinatus on ultrasound was associated with PAR to SAB injection (OR 5.02; *p *< 0.05). Ultrasound evidence of a biceps tendon sheath effusion (OR 8.0; *p *< 0.01) and an intact rotator cuff (OR 1.3; *p *< 0.05) were associated with PAR to GHJ injection. No imaging findings were strongly associated with PAR to ACJ injection (*p *≤ 0.05).

**Conclusions:**

Rotator cuff and SAB pathology were the most common findings on ultrasound and MRA. Evidence of a full thickness supraspinatus tear was associated with symptoms arising from the subacromial region, and a biceps tendon sheath effusion and an intact rotator cuff were associated with an intra-articular GHJ pain source. When combined with clinical information, these results may help guide diagnostic decision making in primary care.

## Background

Shoulder pain is a common and disabling complaint. The reported annual incidence of shoulder pain in primary care is 14.7 per 1000 patients per year [1] with a lifetime prevalence of up to 70% [2]. Recovery from shoulder pain can be slow and recurrence rates are high with 25% of those affected by shoulder pain reporting previous episodes, and 40 to 50% reporting persisting pain or recurrence at 12-month follow-up [3-5].

The most common causes of shoulder pain in primary care are reported to be rotator cuff disorders, acromioclavicular joint (ACJ) disease and glenohumeral joint (GHJ) disorders [6], with classification of these disorders based primarily upon results of clinical tests [1,7-11]. However, inconsistent diagnostic terminology [12], lack of universally accepted diagnostic classification criteria [13,14] and poor specificity of many physical examination tests [15] hamper confidence in classification systems that use clinical test criteria alone.

Diagnostic imaging investigations including shoulder x-ray and diagnostic ultrasound imaging are increasingly being utilised by primary care practitioners to aid diagnosis [16]. More advanced imaging investigations such as magnetic resonance arthrogram (MRA) are also available, providing improved visualisation of pathologies such as glenoid labral lesions and tendon pathology [17]. While previous studies report the prevalence of imaging findings in the general population [18], specific athletic populations [19,20], samples of convenience [21,22] or case-control comparisons for specific shoulder pathology [23], the prevalence of imaged pathology in a prospective cohort of primary care patients suffering a current episode of shoulder pain has not been previously reported. Diagnostic decisions rely upon knowledge of prevalence of a condition in specific populations in order to estimate the likelihood of a positive 'disease' status or outcome following specific tests or investigations [24]. Knowledge of prevalence of imaged pathology in primary care would provide prior probability for specific conditions, thus assisting diagnostic decision-making processes and assessment as to the value of expensive or invasive investigations or interventions.

The interpretation of imaging findings can be complicated by the presence of anatomic variants [25,26] and the high prevalence of asymptomatic pathology especially in ageing populations [18,21]. The prevalence of asymptomatic full-thickness rotator cuff tears more than doubles after the age of 50 years [18], and asymptomatic ACJ arthritis has been identified by magnetic resonance imaging (MRI) in 93% of individuals over the age of 30 years [21]. Despite widespread use of imaging investigations in primary care, the relationship between imaging findings and symptoms has received limited attention. Diagnostic injections of local anaesthetic provide a method for determining whether symptoms arise from a specific structure [27,28]. Following injection of local anaesthetic into an anatomical structure, any subsequent reduction in pain intensity can be measured to assess the likelihood of its involvement in the patient's symptoms [29-31].

The aims of this paper were to report the prevalence of imaged shoulder pathology, and to evaluate the association between imaged pathology and a positive response to diagnostic blocks in a consecutive sample of patients with shoulder pain recruited from a primary care setting.

## Methods

### Study design and setting

The results presented in this paper formed part of a wider prospective, blinded diagnostic accuracy study in which clinical examination and imaging variables (index tests) were compared with results of diagnostic injections of local anaesthetic (reference standard) into the SAB, ACJ and GHJ. Subjects were recruited consecutively from a community-based medical centre and nine physiotherapy practices across Christchurch, New Zealand.

### Ethical approval

The New Zealand Ministry of Health Regional Ethics Committee (Upper South A) granted ethical approval in May 2008.

### Subjects

**Figure 1:**
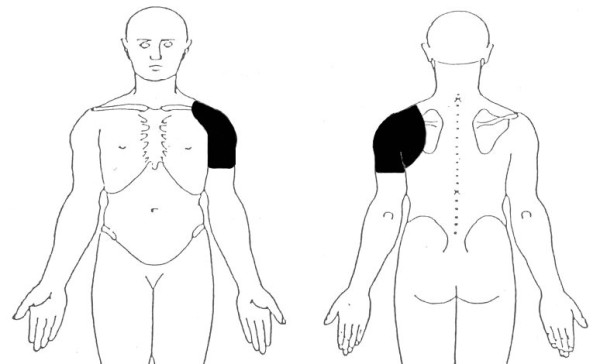
**Distribution of primary pain required for inclusion in the study**.

Consecutive patients presenting to their primary care practitioner (general practitioner (GP) or physiotherapist) for the first time with a new episode of shoulder pain (Figure 1), who were over 18 years of age and able to follow verbal instructions were eligible for inclusion in the study. Exclusion criteria were known fractures or dislocations around the shoulder complex, referred pain from the cervical spine, sensory or motor deficit involving the upper limb, previous surgery to the shoulder or cervical spine or contraindications to imaging or injection procedures.

### Procedures

**Figure 2:**
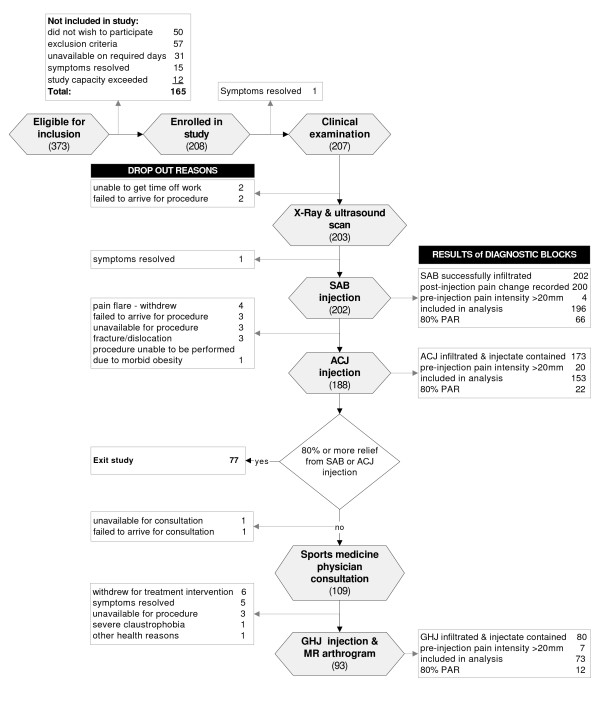
**Diagram showing study procedures, results of diagnostic blocks and dropout explanations**. SAB, subacromial bursa; PAR, positive anaesthetic response (≥80% post-injection reduction in pain intensity); ACJ, acromioclavicular joint; GHJ, glenohumeral joint; MR arthrogram, magnetic resonance arthrogram. Numbers refer to the number (n) of subjects.

Subjects underwent a clinical examination (Additional file_1) followed by a standard shoulder x-ray series, diagnostic ultrasound scan and imaging guided diagnostic injections into the SAB and ACJ. Subjects reporting less than 80% reduction in pain intensity from either of these two injections were reviewed by a sports medicine physician prior to receiving an injection of local anaesthetic into the GHJ, performed as part of a contrast-enhanced MRA procedure. Study procedures are summarised in Figure 2.

### X-ray and diagnostic ultrasound scan

Subjects underwent a standardized series of shoulder radiographs (x-ray) consisting of anterior-posterior (AP) views in neutral, external and internal rotation, axial view and outlet view [32]. X-rays were reported by experienced musculoskeletal radiologists. A standardised report form was used and radiologists recorded specific abnormalities of the ACJ, acromion, GHJ and calcific deposits. Imaging diagnostic criteria are presented in Table 1.

**Table 1:** Imaging diagnostic criteria

Pathology	Imaging Diagnostic Criteria
**X-Ray**	
Acromioclavicular joint	
arthropathy/degenerative change	joint space narrowing, subchondral sclerosis, subchondral cystic change or marginal osteophytes.
osteolysis	bony resorption or increased lucency in distal clavicle.
Glenohumeral joint	
arthropathy/degenerative change	joint space narrowing, subchondral sclerosis, subchondral cystic change or marginal osteophytes.
other	loose bodies, joint calcifications.
Calcification of rotator cuff components	
supraspinatus	calcific deposits adjacent to the greater tuberosity on AP-external rotation x-ray view.
infraspinatus	calcific deposits adjacent to the greater tuberosity on AP-internal rotation x-ray view.
subscapularis	calcific deposits in the anterior shoulder region on axial x-ray view.

**Ultrasound**^**a**^	
ACJ pathology	Capsular hypertrophy, cortical irregularity or osteophytes, capsular bulge, joint space narrowing or widening.
Glenohumeral joint effusion	more than 2 mm between posterior glenoid labrum and posterior capsule.
Rotator cuff	
normal	normal contour, normal echogenicity.
calcification	focal increase in echogenicity with or without shadowing.
tendinosis	tendon thickening or decreased echogenicity.
tear	
intrasubstance	hypoechoic change not extending to articular or bursal surface.
partial thickness	SSp and ISp: hypoechoic change extending to either the articular or bursal surface. Subscapularis: partial fibre discontinuity.
full thickness	SSp and ISp: hypoechoic region extends from bursal to articular surface. Subscapularis: complete fibre discontinuity.
Subacromial bursa	
bursitis	hypoechoic fluid or effusion present and >1 mm thick.
bursal thickening	≥2 mm measured from deep margin of deltoid to superficial margin of supraspinatus.
"bunching"	Fluid distension of the SAB or 'buckling' of the rotator cuff during abduction

**MR arthrogram**^**a**^	
Acromioclavicular joint	
arthropathy/degenerative changes	capsular hypertrophy with or without joint space narrowing, subchondral cystic change, bone marrow oedema or osteophytes
osteolysis	bony resorption or bone marrow oedema in the distal clavicle
Rotator cuff	
normal	normal contour, normal signal
tendinosis	tendon thickening or mild increase in T2 signal
intrasubstance tear	linear increase in T2 signal which does not extend to the articular or bursal surface.
partial thickness tear	linear increase in T2 signal extending to the (bursal or articular) margins.
full thickness tear	fluid signal intensity or contrast extending from the bursal to the articular side lesion of the rotator cuff. Contrast seen in the SAB.
Subacromial bursitis	increased T2 signal within the SAB
Glenohumeral joint	
rotator interval pathology	thickening, signal change or tear involving the biceps pulley, superior glenohumeral or coracohumeral ligament, or synovitis in the rotator interval.
arthropathy/degenerative change	chondral loss, subchondral sclerosis, cystic changes, bone marrow oedema or osteophytes
labral tear	contrast extending into- or undermining the glenoid labrum, not conforming to normal variant anatomy.

Abbreviations: AP, antero-posterior view; ACJ, acromioclavicular joint; SSp, supraspinatus; ISp, infraspinatus; SAB, subacromial bursa;

^a^definitions based upon accepted diagnostic criteria [33,35]

Diagnostic ultrasound scans were performed by trained and experienced musculoskeletal sonographers and reported by fellowship trained musculoskeletal radiologists. Examinations were performed using a Philips IU22 machine with a 5-12 MHz linear array probe using a standardised scan procedure [33,34]. The scan procedure is described in Additional file_2.

The SAB was observed during dynamic abduction and 'bunching' under the acromion and the coracoacromial ligament (CAL) was recorded. Subacromial bursal dimensions were measured from the deep margin of deltoid muscle to superficial margin of supraspinatus tendon in all cases where this distance was measurable (dimensions exceeding 1 mm).

### Diagnostic injections

#### Subacromial bursa injection

Subjects were positioned supine with the arm in external rotation. Under aseptic conditions, a 22-gauge needle was used to inject 5 mL of 1% lidocaine hydrochloride (xylocaine™) into the SAB under ultrasound guidance using an anterior approach. When needle placement inside the SAB was confirmed by ultrasound, the contents of the syringe were emptied into the bursa. The radiologist recorded whether the SAB was successfully infiltrated. A video of this procedure may be viewed in Additional file 3_SAB injection, compatible with Windows^® ^Media Player software.

#### Acromioclavicular joint injection

One week after the SAB injection, local anaesthetic was injected into the ACJ under fluoroscopic guidance using contrast enhancement. Subjects were positioned supine with the arm in external rotation. Under aseptic conditions, a 22-gauge needle was inserted into the ACJ using a direct anterior approach. Iodinated contrast (0.5 ml of Omnipaque 300 GE Healthcare) was introduced and fluoroscopic images used to confirm needle placement within the ACJ. Approximately 2 mL of 1% lidocaine hydrochloride (xylocaine™) was then injected into the joint. The radiologist recorded whether the ACJ was successfully infiltrated and whether the injectate was contained within the joint. A video of this procedure may be viewed in Additional file 4_ACJ injection.

#### Glenohumeral joint injection

Approximately one week after the ACJ injection, subjects reporting less than 80% relief from both the SAB and ACJ injections underwent a GHJ arthrogram and intra-articular injection of local anaesthetic and gadolinium prior to magnetic resonance imaging (MRI). Subjects were positioned supine and the GHJ injection carried out under fluoroscopic guidance as described for the ACJ injection (above) using 5 mL of iodinated contrast. A mixture of 0.5 mL gadolinium (0.5 mmol/ml Gd-DOTA Guerbet France) and 10 mL 1% lidocaine hydrochloride (xylocaine™) was injected into the joint. The radiologist recorded whether the injectate was contained within the joint. A video of this procedure may be viewed in Additional file 5_GHJ injection.

#### Determination of post-injection change in pain intensity

Immediately prior to each injection, all subjects were examined using up to six clinical tests identified as being provocative of the subjects typical symptoms during the initial clinical examination (Additional file_1). Pre-injection pain intensity was recorded for each clinical test on a 100 m visual analogue scale (VAS) where 0 mm indicated "no pain" and 100 mm represented "worst imaginable pain". Tests were repeated between 5 and 15 minutes following each injection and post-injection pain intensity VAS scores recorded again. The percentage change in pain intensity (anaesthetic response) was calculated for each test [(post-injection VAS - pre-injection VAS/pre-injection VAS)*100]. The average percent change from all tests was then calculated. A post-injection reduction in pain intensity of 80% or more was used as the criterion for a positive anaesthetic response (PAR). Subjects who did not reach an average of 80% pain relief following the SAB and ACJ injection were evaluated by a sports medicine physician and referred for the MRA investigation.

### Magnetic resonance arthrogram imaging

Magnetic resonance imaging was obtained within 30 minutes of the GHJ injection. Imaging was performed with 3.0 Tesla General Electric-Milwaukee (GE) Signa HDxt platform running version 15 software. A conventional MR arthrography protocol was followed (Additional file_2) [35].

### Blinding

The investigator performing the clinical examination and pre- and post-injection clinical tests (AC) was blinded to all diagnostic and treatment information from referring practitioners and to results of imaging procedures. Sonographers and radiologists were blinded to all clinical information prior to the x-ray, ultrasound scans and MRA procedure, and were blinded to results of anaesthetic response to injections.

### Sample size considerations

Sample size was estimated using methods described by Flahault et al., (2005) [36]. Sample size was calculated for the diagnostic sub-group with the lowest expected prevalence (ACJ). The minimal acceptable lower confidence limit was set at 0.75 and expected sensitivity/specificity were both set at 0.90. A review of sample size estimates after the first 100 cases indicated lower than expected prevalence of PAR to ACJ diagnostic block and sample size was adjusted in order to maintain precision of diagnostic estimates.

### Statistical analysis

The prevalence of imaged pathology and response to each of the diagnostic blocks are reported as frequency and percentages. Contingency tables (2 × 2) were constructed and Fishers exact test was used to compare proportions of positive and negative imaging findings in the anaesthetic response groups for each diagnostic injection procedure. *P*-values of ≤0.05 were used to indicate statistical significance. Odds ratios (OR) and 95% confidence intervals (CI) for PAR to diagnostic blocks were calculated. Statistical Package for the Social Sciences (SPSS) version 17.0 (IBM^® ^Corporation 2010) was used for the analysis.

Due to the known limitations of VAS scales for measuring change in pain intensity when pre-injection pain levels are low (<20 mm) [37], only cases where pre-injection pain intensity exceeded 20 mm were included in the analysis of anaesthetic response to diagnostic injections. Average percent change in pain intensity was calculated for the index tests with positive integers indicating increased post-injection pain intensity, and negative integers indicating decreased post-injection pain intensity.

## Results

### Subjects

A total of 208 subjects were included in the study between July 2009 and June 2010. Details of progression of subjects through the study and dropout explanations are presented in Figure 2. Demographic information for those included in the study is presented in Table 2. There were no significant differences between those included and excluded from the study with respect to age or gender. Symptom duration was shorter (median 2 weeks; IQ range 4 weeks) in subjects excluded from the study (Mann-Whitney *p *< 0.001). There were no significant differences in demographic characteristics between the total sample and the sub-group who received the GHJ injection as part of the MRA procedure (*p *> 0.05).

**Table 2:** Subject demographics

	All subjects (n = 208)		MRA group (n = 93)	
	
**Subject characteristics**	Mean (SD)	Range	Mean (SD)	Range
Age (years)	42 (14)	18-81	42 (14)	18-81
Height (cm)	172 (10)	147-199	172 (10)	151-198
Weight (kg)	80.6 (18.0)	50.3-189.0	82.3 (15.8)	52.7-125.3
Symptom duration (weeks)*	7 (13)	0-175	7 (13)	0-175
Worst pain previous 48 hours (100 mm VAS)	62 (23)	3-100	63 (24)	3-100
Average pain previous 48 hours (100 mm VAS)	37 (22)	1-100	37 (24)	1-100
	**n (%)**		**n (%)**	

Male gender	107 (51)		53 (57)	
Right hand dominant	110 (53)		79 (85)	
Dominant arm affected	110 (53)		48 (52)	
ACC Claim	193 (93)		86 (93)	
Referrals				
physiotherapist	203 (98)		89 (96)	
general practitioner	5 (2)		4 (4)	
Employment status				
in paid employment	166 (80)		76 (82)	
on modified duties due to shoulder pain	18 (9)		10 (11)	
off work due to shoulder pain	7 (3)		4 (4)	
not currently employed/working	41 (20)		17 (18)	
Co-existent medical conditions	70 (34)		33 (36)	
Current smoker	39 (19)		18 (20)	

Abbreviations: MRA, magnetic resonance arthrogram; SD, standard deviation; VAS, visual analogue scale: ACC, Accident Compensation Corporation.

*symptom duration was not normally distributed. Figures presented are median (IQ range).

### Prevalence of imaged pathology

#### X-ray and ultrasound scan

**Figure 3:**
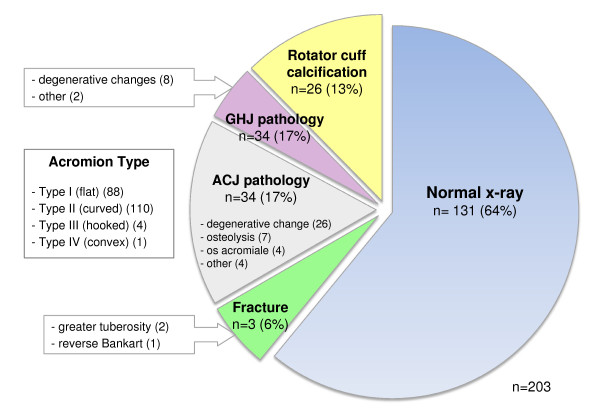
**Prevalence of pathology identified on x-ray**. n, number of cases; ACJ, acromioclavicular joint; GHJ, glenohumeral joint

**Figure 4:**
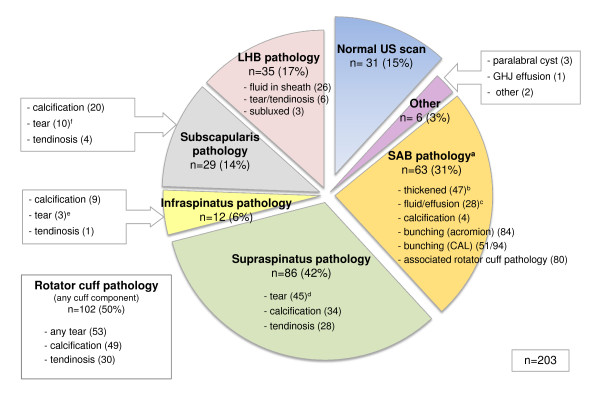
**Prevalence of pathology identified on ultrasound scan**. (n), number of cases; US, ultrasound; GHJ, glenohumeral joint; SAB, subacromial bursa; CAL, coracoacromial ligament; LHB, long head of biceps tendon. ^a^Subacromial pathology: any one of three present; dimension ≥2 mm, fluid/effusion or calcification. ^b^Subacromial bursa dimensions: <1 mm (71); 1-2 mm (82); 2-3 mm (42); >3 mm (5). ^c^Subacromial bursal effusion associated with full thickness rotator cuff tear (7). ^d^Supraspinatus tears: intrasubstance (23); partial thickness-bursal surface (4); partial thickness-articular surface (8); full thickness (10). ^e^Infraspinatus tears: intrasubstance (1); partial thickness (1); full thickness (1). ^f^Subscapularis tears: intrasubstance (5); partial thickness (4); full thickness (1).

**Figure 5:**
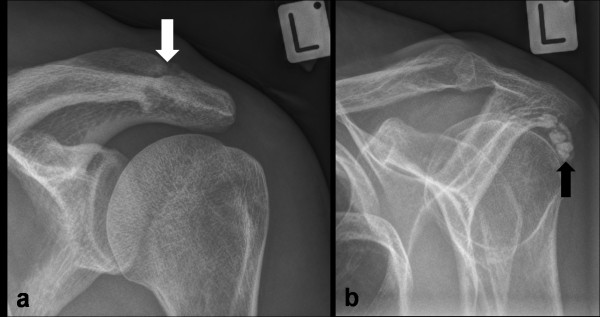
**Shoulder x-ray images of ACJ pathology and rotator cuff calcification**. a) AP x-ray view in external rotation showing degenerative acromioclavicular joint changes (white arrow); b) outlet view showing calcification in line with the infraspinatus tendon (black arrow).

The prevalence of the pathologies identified on x-ray and ultrasound are presented in Figures 3 and 4. Acromioclavicular joint (Figure 5a) and GHJ pathology were the most common x-ray findings (both 17%) and calcification involving the rotator cuff was reported in 13% of subjects (Figure 5b).

**Figure 6:**
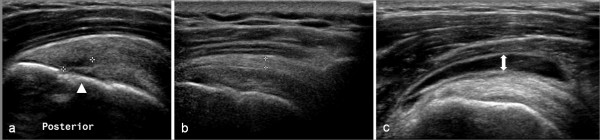
**Ultrasound scan images of subacromial bursa and supraspinatus pathology**. a) hypoechoic region (between calipers) indicating an intrasubstance tear within posterior fibres of supraspinatus (longitudinal view) overlying the head of humerus (white arrowhead); b) thickened subacromial bursa (calipers); c) bunching of the SAB (white arrow) under the acromion during dynamic abduction.

Rotator cuff pathology was the most prevalent pathology on ultrasound (50%), with supraspinatus the most commonly affected rotator cuff component, accounting for 86 of the 102 cases (85%) of rotator cuff pathology. Tears were the most common pathology affecting supraspinatus accounting for 52% of all supraspinatus pathology and intrasubstance tears were the most common type of tear accounting for 51% of all supraspinatus tears (Figure 6a). Calcification was the most common finding in infraspinatus (59%) and subscapularis (69%) compared with 39% in supraspinatus.

**Figure 7:**
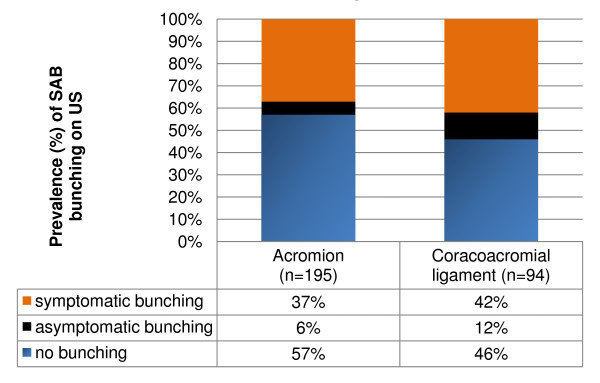
**Prevalence of subacromial bursa bunching under the acromion and coracoacromial ligament on ultrasound during dynamic abduction**. SAB, subacromial bursa; US, ultrasound; CAL, coracoacromial ligament. Percentages are in reference to the number of cases in which bursal bunching was assessed (acromion n = 195; CAL n = 94).

Prevalence of SAB pathology was 31% and bursal thickening (dimensions exceeding 2 mm) was reported in 23% of subjects (Figure 6b). Bunching of the SAB under the acromion was observed in 84 subjects (43%) (Figure 6c), and this was associated with reproduction of symptoms in 72 subjects (86% of cases in which bunching was observed). Bunching under the CAL was observed in 51 of the 94 cases (54%) in which this was assessed, and was associated with reproduction of symptoms in 40 subjects (78% of cases in which bunching was observed) (Figure 7).

#### Magnetic resonance arthrogram

**Figure 8:**
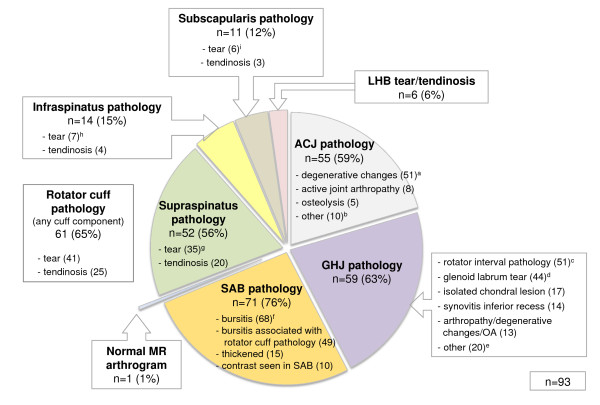
**Prevalence of pathology identified on MR arthrogram**. (n), number of cases; LHB, long head of biceps tendon; ACJ, acromioclavicular joint; GHJ, glenohumeral joint; OA, osteoarthritis; SAB, subacromial bursa; ^a^ACJ degenerative changes: mild (28); moderate (18); severe (5). ^b^Acromioclavicular joint pathology - other: os acromiale (2); unfused acromial ossification centre (1); acromial spur (4); widened joint space/subluxation (2); synovitis (1). ^c^Rotator interval pathology: coracohumeral or superior glenohumeral ligament thickening (40); rotator interval synovitis (39); biceps pulley, coracohumeral or superior glenohumeral ligament tear (13). ^d^Glenoid labrum tear: isolated labral tear (5); associated pathology present (39); SLAP tear (20); SLAP Type II (17), Type III (2), Type IV (1); anterior-inferior tear (9); semi- or full circumferential tear (7); posterior-superior tear (1); other tear (9); paralabral cyst (10); paralabral cyst causing suprascapular nerve compression (2). ^e^Glenohumeral joint pathology - other: bony irregularity humeral head without marrow oedema (12); Hill-Sachs lesion (3); intra-articular/osseous body (3); ganglion cyst between coracoacromial and coracohumeral ligaments (1); greater tuberosity fracture (1). ^f^Subacromial bursitis: mild (52); moderate (12); severe (4) ^g^Supraspinatus tears: intrasubstance (11); partial thickness-bursal surface (5); partial thickness articular surface (12); full thickness (7). ^h^Infraspinatus tears: intrasubstance (4); partial thickness (3); full thickness (0) ^i^Subscapularis tears: intrasubstance (4); partial thickness (0); full thickness (2)

**Figure 9:**
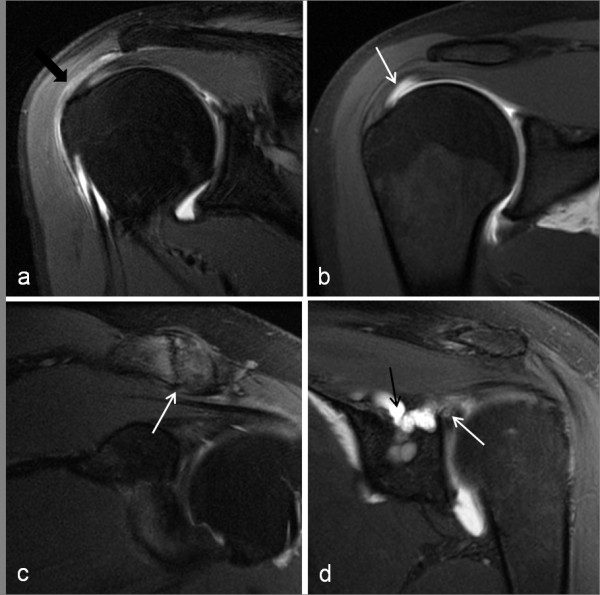
**MR arthrogram images of shoulder pathology**. a) subacromial bursitis - coronal PD fat saturated image showing region of hyperintensity in the subacromial bursa (black arrow); b) partial thickness, articular surface supraspinatus tear (white arrow) - coronal T1 fat saturated image showing contrast extending into the supraspinatus tendon. c) ACJ degenerative changes (white arrow) -coronal PD fat saturated image; d) type III SLAP tear (white arrow) with contrast filling a paralabral cyst (black arrow) which extended into the supraglenoid and suprascapular notch causing neural compression -coronal PD fat saturated image.

The prevalence of MRA findings is shown in Figure 8. Only one case was reported as "normal" (no abnormality reported) and 74% of cases demonstrated multiple pathologies. The most commonly reported MRA finding overall was SAB pathology (76%) with subacromial bursitis reported in 68 subjects (73%) (Figure 9a). Rotator cuff pathology affected at least one of the rotator cuff components in 65% of cases. Supraspinatus was the most frequently affected component of the rotator cuff (85% of all rotator cuff pathology) and tears were the most common pathological finding in all rotator cuff components accounting for 41 of the 61 cases (67%) of rotator cuff pathology. Partial thickness tears involving the articular surface were the most common type of supraspinatus tear identified (34% of all supraspinatus tears) (Figure 9b). GHJ pathology (63%) and ACJ pathology (59%) were also highly prevalent with rotator interval pathology (GHJ) and degenerative ACJ changes (Figure 9c) (both 55%) the most common findings. Glenoid labrum tears were present in 47% of all subjects who received the MRA and were associated with paralabral cysts in 10 cases (23%). Suprascapular nerve compression was associated with paralabral cysts in two cases (2%) (Figure 9d).

### Prevalence of anaesthetic response to diagnostic blocks

**Figure 10:**
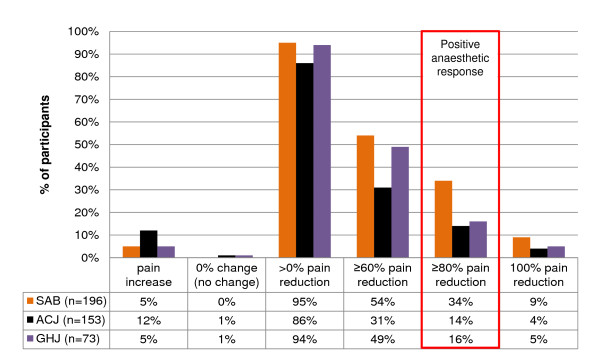
**Anaesthetic responses to diagnostic blocks**. SAB, subacromial bursa; ACJ, acromioclavicular joint; GHJ, glenohumeral joint.

The anaesthetic response profiles for the diagnostic injections are presented in Figure 10. There were no observable differences in the frequency of imaged pathology between those in whom post-injection pain intensity increased compared with cases in which a post-injection decrease in pain was reported. Results for the injection procedures are presented in Figure 2. Infiltration of the SAB was confirmed in all cases and a PAR (≥80% pain relief) was reported by 66 subjects (34%) following the SAB injection. Average ACJ injection volume was 2.1 mL (SD 0.7 mL) and 22 of the 153 subjects (14%) in whom the injectate was contained within the ACJ and whose pre-injection pain intensity exceeded 20 mm on the 100 mm VAS scale reported an 80% PAR. Ninety three subjects received the GHJ injection as part of the MR arthrogram procedure and an 80% PAR was reported by 12 of the 75 subjects (16%) in whom the injectate was contained within the GHJ and pre-injection pain intensity exceeded 20 mm.

### Association between imaged pathology and response to diagnostic blocks

Imaging variables associated with PAR to diagnostic block (*p *≤ 0.05) and demonstrating a magnitude of association OR greater than 2.0 are summarised in Table 3. Results for all other x-ray and ultrasound variables are presented in Additional file_6 (SAB and ACJ injection) and Additional file_7 (GHJ injection). Results for all other MRA variables are presented in Additional file_8.

**Table 3:** Summary of imaging variables demonstrating association with positive anaesthetic response to diagnostic blocks (*p *≤ 0.05 or OR >2.0)

Pathology identified on imaging	Pathology identified (total cases) (n)	% with pathology present reporting PAR	% with pathology absent reporting PAR	OR (95% CI)	Fishers test (*p *value)
**SAB injection **(PAR n = 66)					
X-ray: type 3 acromion	4	75	33	6.2 (0.64, 61.23)	0.109
X-ray: os acromiale	4	75	33	6.1 (0.63, 60.25)	0.112
X-ray: supraspinatus calcification	16	56	31	2.8 (1.00, 7.97)	0.054
US: supraspinatus calcification	33	49	31	2.1 (1.00, 4.55)	0.068
US: supraspinatus FTT	10	70**	32	5.0 (1.25, 20.11)	0.033

**ACJ injection **(PAR n = 22)					
X-ray: ACJ pathology	21	14	16	2.1 (0.69, 6.52)	0.189
US: supraspinatus tear PTT (articular surface)	8	0	17	2.1 (0.39, 11.05)	0.323
US: LHB tendinosis	3	0	16	3.1 (0.27, 35.39)	0.374

**GHJ injection **(PAR n = 12)					
US: no rotator cuff tear	19	21**	0	1.3 (1.11, 1.46)	0.029
US: supraspinatus tendinosis	11	27	14	2.3 (0.51, 10.30)	0.374
US: subscapularis tendinosis	3	33	15	2.8 (0.23, 33.27)	0.421
US: biceps tendon sheath effusion	13	46**	10	8.0 (2.02, 31.72)	0.004
MRA: ACJ pathology	46	20	11	2.0 (0.50, 8.23)	0.516
MRA: osteolysis lateral clavicle	5	40	15	3.9 (0.58, 26.58)	0.187
MRA: contrast seen in SAB	6	33	15	2.9 (0.47, 17.99)	0.254

Abbreviations: PAR, positive anaesthetic response (≥80% post-injection pain intensity reduction); OR, adjusted odds ratio; CI, confidence interval; SAB, subacromial bursa; US, ultrasound; FTT, full thickness tear; ACJ, acromioclavicular joint; PTT, partial thickness tear; LHB, long head of biceps; GHJ, glenohumeral joint; MRA, magnetic resonance arthrogram.

Percentages do not total 100% as these represent proportion of subjects with or without pathology on imaging (row percentages in contingency table) in the PAR group. Negative anaesthetic response group results (column percentages) are not presented.

**p ≤ 0.05

A full thickness supraspinatus tear identified by ultrasound imaging was associated with PAR to SAB injection (OR 5.0, *p *≤ 0.05). None of the imaging variables were strongly associated with PAR to ACJ injection (*p *> 0.05). The strongest association of any imaging variable with diagnostic block was the association between biceps tendon sheath effusion identified on ultrasound and PAR to GHJ injection (OR 8.0; *p *< 0.01). A tear of the rotator cuff reported on ultrasound was negatively associated with a PAR to GHJ injection (*p *< 0.05). When recoded, an 'intact' rotator cuff on ultrasound demonstrated an OR of 1.3 for a PAR.

## Discussion

This is the first report of the prevalence of imaged pathology and anaesthetic responses to diagnostic injection into the SAB, ACJ and GHJ in a sample of primary care patients with shoulder pain. Estimates of the likelihood of symptomatic pathologies being present that affect these sites will increase or decrease as details from the history and physical examination are added to the imaging findings, but prior probability (prevalence) of these conditions in the population of interest is the necessary baseline and starting point [24]. This study provides the prior probability data for specific pathologies and pain sources at the 80% pain reduction level in a sample of primary care patients. This knowledge may help inform clinical decisions regarding treatment interventions, the use of advanced imaging or specialist referral.

### Prevalence of imaged pathology

#### X-ray and diagnostic ultrasound scan

Shoulder x-rays were reported as 'normal' in 64% of cases however the detection of three unsuspected fractures in our study population highlights the use of x-ray as a valuable screening tool. The prevalence of calcification identified on x-ray (13%) was similar to previous reports (10%) [38].

Subacromial bursa pathology was a common ultrasound finding (31%) in our symptomatic sample. We used the criterion of bursal dimension ≥2 mm, calcification or bursal fluid or effusion or to classify 'SAB pathology'. Opinions vary regarding the dimension (thickness) at which the normally thin hypoechoic line of the SAB is regarded as pathological. Some have suggested the ability to view and measure the SAB at all represents pathological thickening [39], others consider more than 2 mm thickness to be pathological [40-42] and some suggest SAB thickness compared with the unaffected side irrespective of bursal dimension to be of more clinical relevance [43]. Recent theories question whether SAB thickening is even pathological, proposing it may be the result of adaptation to repeated overhead activity [16]. Variable agreement (kappa 0.50 to 0.89) has also been reported between musculoskeletal ultrasound experts for identification of SAB pathology on ultrasound [44-47] with most disagreements relating to variations in dynamic assessment and judgement of SAB fluid as being normal or pathological [47]. Technicalities surrounding the ultrasound diagnosis of SAB pathology, lack of expert consensus upon the dimension at which the SAB is considered pathological and the poor understanding of the relationship between SAB histopathology and imaging findings mean that the reported prevalence of SAB pathology on ultrasound is likely to vary. Bursal bunching was also identified in a high proportion of subjects, however bunching was asymptomatic in 14% (acromion) and 22% (CAL) of cases in which bunching was observed. This highlights the need to correlate imaging findings with clinical symptoms when considering the diagnosis of 'subacromial impingement'.

#### Magnetic resonance arthrogram

Magnetic resonance arthrogram findings in the subgroup of subjects receiving the investigation, revealed a high prevalence of multiple pathologies (74%), similar to previous reports (77%) in an asymptomatic primary care population [48]. In the subjects who received the MRA, SAB and ACJ pathology were reported respectively in 76% and 59% of subjects, all of whom had previously been classified as 'non-responders' at the 80% pain relief level following injection of local anaesthetic into these structures. Marrow oedema on MRI has been reported as a reliable indicator of symptomatic ACJ pathology [23]. Our study identified eight cases (9%) of active ACJ arthropathy with marrow oedema in subjects who had previously demonstrated a NAR to ACJ injection, however the inability of the local anaesthetic to penetrate to the level of subchondral bone, thereby classifying those subjects as 'non-responders' to ACJ injection, represents a likely explanation for this result.

Rotator cuff pathology was reported in more than half of subjects on both ultrasound and MRA with rotator cuff tears identified in 26% and 44% of subjects with the respective imaging procedures. Although no primary care imaging studies are available for direct comparison, these results are similar to previous reports of the prevalence of rotator cuff tears in asymptomatic populations on ultrasound [18] and MRI [48]. Of interest was the higher number of intrasubstance tears involving infraspinatus, and partial thickness (articular surface) supraspinatus tears identified on MRA compared with the number identified on ultrasound imaging, despite the smaller sample number in this subgroup. While identification of an intrasubstance tear on MRA is unlikely to alter management at primary care level unless it is associated with more serious pathology, partial thickness tears of the rotator cuff are reported to be of prognostic significance due to the high proportion that increase in size or progress to full thickness tears if left untreated [49]. Ultrasound imaging has previously demonstrated only moderate pooled sensitivity (72%) for detection of partial thickness rotator cuff tears compared with MRI or surgery [50]. Variable agreement among experts on the presence of partial thickness rotator cuff tears on ultrasound (kappa 0.63; 88% to 92% agreement) has also been reported [44,47,51]. Results of MRI scans have been shown to alter clinical decisions regarding management of rotator cuff tears in the orthopaedic setting [52] and MRA may therefore be indicated at the primary care level if there is clinical suspicion of rotator cuff disruption in the presence of equivocal ultrasound findings.

The prevalence of intra-articular GHJ pathology on MRA in this sub-group of subjects was also high (63%) with rotator interval pathology (55%) and glenoid labral tears (47%) the most common findings. However, despite the high prevalence of GHJ pathology in this study, only 16% of individuals were classified as responders to the GHJ injection at the 80% pain relief level. During the MRA procedure, contrast was introduced into the GHJ through the region of the rotator interval and in some subjects the appearance of contrast in this region on subsequent MRI films may have been difficult to distinguish from mild rotator interval pathology. Glenoid labral tears are frequently associated with other extra-articular pathology such as rotator cuff tears [53-56], and the rotator interval also has complex pathoanatomic relationships with supraspinatus, subscapularis and the long head of biceps tendon [57]. The high proportion of multiple pathology and low GHJ PAR rate in this study may be partially explained by the concurrent involvement of extra-articular structures.

### Association between imaging findings and anaesthetic response

Subjects with full thickness tears of supraspinatus identified by ultrasound imaging were more likely to experience a PAR to SAB injection than those without a full thickness tear. Full thickness supraspinatus tears affect the SAB-rotator cuff interface and infiltration of the torn cuff with anaesthetic through this disruption is the likely explanation for this finding. The small proportion of PAR among those with an intrasubstance supraspinatus tear (intact margins) reported on ultrasound supports this theory, however none of the four cases in which bursal-surface supraspinatus tears were identified were classified as responders to the SAB injection. None of the imaging variables were strongly associated with PAR to ACJ injection. The high prevalence of asymptomatic degenerative changes particularly in individuals older than 30 years (93%) [21] may explain this result.

A long head of biceps tendon sheath effusion on ultrasound was significantly related to a PAR to GHJ injection. The biceps tendon sheath is a synovial extension of the GHJ capsule and may therefore be indicative of a GHJ effusion resulting from intra-articular GHJ pathology or systemic inflammatory disease. A biceps tendon sheath effusion on ultrasound has been shown to be more sensitive than arthrography for detection of intra-articular GHJ pathology [58]. It is also a common finding in those suffering rheumatoid arthritis [59,60] and has been found to be predictive of degenerative GHJ arthritis and polymyalgia rheumatica [53,61]. In the current primary care study, half the subjects with a biceps tendon sheath effusion reported on ultrasound were classified as positive 'responders' to the GHJ diagnostic block at the 80% pain reduction standard. The likely explanation for the PAR is the anaesthetisation of synovial tissue within the GHJ. Although this finding may implicate an intra-articular pain source, it is a non-specific result and further imaging investigations such as MRI or laboratory tests would be required to identify the specific pathology responsible for the synovial effusion. The magnitude of association of the biceps tendon sheath effusion on ultrasound with PAR to GHJ injection seen in this study (OR 8.00), and a lower 95% confidence limit of 2.0 suggest this finding may be of value in the primary care setting when considering further imaging investigation, laboratory testing or referral for higher levels of care.

Subjects with an intact rotator cuff on ultrasound also demonstrated a higher proportion of PAR to GHJ injection (*p *< 0.05) than those in whom a rotator cuff tear was identified. This could imply that in subjects with a rotator cuff tear, the tear itself may have been more symptomatic than any co-existent intra-articular GHJ pathology resulting in the NAR to GHJ diagnostic block. Although the OR for PAR to GHJ injection in the presence of an intact rotator cuff on ultrasound was small (1.27), the CI did not include 1.0, and could represent a clinically meaningful increase in the likelihood of a PAR since the prevalence of this imaging finding was high (74%) [62]. Current guidelines advocate ultrasound imaging only when a major rotator cuff tear is suspected when surgery may be considered as a treatment option [63]. However, these results may provide additional justification for the use of diagnostic ultrasound imaging in the primary care setting to inform decisions regarding further investigations for intra-articular GHJ pathology in the presence of an intact rotator cuff and relevant clinical findings.

#### Limitations of the study

The definition of 'accident' in the context of subject 'claim status' in this study is influenced by New Zealand's' unique Accident Compensation Corporation legislation. Although the majority of subjects included in our study had a current ACC claim, this does not necessarily imply a significant degree of trauma, and complaints included many less severe conditions with low levels of functional disability. Those whose shoulder pain is not covered by an ACC claim may, however, be less likely to present for medical assessment and may be under-represented in this study. Due to the cost of the MRA procedures it was not possible for every subject to undergo this procedure, and several subjects with high and low levels of pain intensity withdrew from the study prior to the MRA representing a potential source of selection bias in this subgroup of subjects.

## Conclusions

Rotator cuff and SAB pathology were the most common findings on both ultrasound and on MRA in this primary care cohort. A full thickness supraspinatus tear on ultrasound was associated with subacromial pain according to our criterion, and ultrasound findings of a biceps tendon sheath effusion and an intact rotator cuff were associated with pain arising from the GHJ in a subgroup of subjects. Results provide the prior probability of imaged pathology, and when combined with clinical examination findings may inform decisions in primary care regarding treatment interventions and the need for advanced diagnostic imaging or specialist referral.

## Competing interests

The authors declare that they have no competing interests.

## Authors' contributions

All authors were involved with conception and design of the study. AC performed all the clinical examination and pre-and post-injection clinical tests, collected and managed all data, carried out the preliminary analysis and drafted the manuscript. MC was involved in selection of guided diagnostic block and imaging procedures, performed and reported MRA procedures and provided radiological guidance in interpretation and discussion of results. ML, WH and PM contributed to methodological development, interpretation of data and critical appraisal of the manuscript for academic and clinical content. All authors read and approved the final manuscript.

## Authors' information

This research was conducted as part of a larger diagnostic accuracy study which is the topic of AC's PhD thesis being conducted through AUT University, Auckland, New Zealand. Separate manuscripts are in preparation that will report the results of diagnostic accuracy calculations and the predictive ability of clinical examination and imaging findings to identify pain arising from specific structures, and specific shoulder pathology.

## Consent statement

Written informed consent was obtained from the patient for publication of this case report and accompanying images. A copy of the written consent is available for review by the Editor-in-Chief of this journal.

## Pre-publication history

The pre-publication history for this paper can be accessed here:

http://www.biomedcentral.com/1471-2474/12/119/prepub

## Supplementary Material

Additional file 1 **Clinical examination procedures**. Table listing the clinical examination procedures used from which pre-injection provocative clinical tests were identified. Click here for file

Additional file 2 **Diagnostic ultrasound and magnetic resonance arthrogram procedures**. Description of the diagnostic ultrasound and magnetic resonance arthrogram imaging protocols used in this study. Click here for file

Additional file 3 **Subacromial bursa injection procedure**. Video file showing ultrasound guided injection of local anaesthetic into the subacromial bursa. Click here for file

Additional file 4 **Acromioclavicular joint injection procedure**. Video file showing injection of local anaesthetic into the acromioclavicular joint under fluoroscopic guidance. Click here for file

Additional file 5 **Glenohumeral joint injection procedure**. Video file showing injection of local anaesthetic into the glenohumeral joint under fluoroscopic guidance. Click here for file

Additional file 6 **Association between x-ray and ultrasound variables and positive anaesthetic responses to subacromial bursa and acromioclavicular joint diagnostic blocks**. Table showing additional results for x-ray and ultrasound imaging variables that were not associated with positive anaesthetic responses to subacromial bursa and acromioclavicular diagnostic blocks. Click here for file

Additional file 7 **Association between x-ray and ultrasound variables and positive anaesthetic responses to glenohumeral joint diagnostic block**. Table showing additional results for x-ray and ultrasound imaging variables that were not associated with positive anaesthetic response to glenohumeral joint diagnostic block. Click here for file

Additional file 8 **Association between magnetic resonance arthrogram variables and positive anaesthetic responses to glenohumeral joint diagnostic block**. Table showing additional results for magnetic resonance imaging variables that were not associated with positive anaesthetic response to glenohumeral diagnostic block. Click here for file
