# Supplementary material for: A prospective study of shoulder pain in primary care: Prevalence of imaged pathology and response to guided diagnostic blocks
Source: BMC Musculoskelet Disord. 2011 May 28;12:119. doi: 10.1186/1471-2474-12-119 (PMC3127806; doi:10.1186/1471-2474-12-119)
Supplement: Additional file 1 — Clinical examination procedures. Table listing the clinical examination procedures used from which pre-injection provocative clinical tests were identified. [file 1471-2474-12-119-S1.PDF]

Additional file_1: Clinical examination variables

Clinical examination variables
**History**
Medical and smoking history
Occupation: status and demand
Past history of shoulder pain
Family history of shoulder pain
Mechanism and date of onset
Pain location
Nature of pain constant/intermittent
Pain severity (best, average and worst)
Pain aggravated by overhead activity
Night pain disturbs sleep
**Physical Examination**
Observation
Cervical spine pain with movement testing
Painful arc abduction
Active elevation (flexion)
Hand-behind-back
Scapula movement tests reproduced symptoms
Resisted tests (abduction, external & internal rotation)
Passive ROM (GHJ abduction, external & internal rotation, cross-body adduction)
Palpation (typical pain produced)
Orthopaedic tests
Hawkins-Kennedy test
drop-arm test
empty can test
active compression test
external rotation lag sign
belly-press test
Speeds test
biceps load II test
apprehension/relocation test
