# Supplementary material for: A prospective study of shoulder pain in primary care: Prevalence of imaged pathology and response to guided diagnostic blocks
Source: BMC Musculoskelet Disord. 2011 May 28;12:119. doi: 10.1186/1471-2474-12-119 (PMC3127806; doi:10.1186/1471-2474-12-119)
Supplement: Additional file 2 — Diagnostic ultrasound and magnetic resonance arthrogram procedures. Description of the diagnostic ultrasound and magnetic resonance arthrogram imaging protocols used in this study. [file 1471-2474-12-119-S2.PDF]

Additional file_2. Diagnostic imaging procedures

Diagnostic ultrasound procedure:
A standardised procedure was performed as follows, with the patient sitting: 1) palm face up on their knee (long head of biceps tendon); 2) elbow tucked into their side with external rotation of the shoulder (subscapularis); 3) arm resting on lap in neutral rotation with elbow behind body (supraspinatus); 4) hand in the small of the back with palm facing outwards to visualize (supraspinatus); 5) hand placed on the opposite shoulder (infraspinatus, ACJ, posterior labrum and glenohumeral joint). Scanning was conducted along the line of each tendon and at 90 degrees to the tendon.

Magnetic resonance arthrogram protocol:
The patient was positioned supine with the affected arm extended alongside their body and externally rotated. Total scan time was 30 minutes including patient positioning.

Series 1: Axial oblique (Obl) T1 (Time to Echo (TE)/Time to Repeat (TR) Min full/640, Echo Train Length (ETL) 4, receive bandwidth (BW) 41.67, slice thickness (ST)/slice gap (SG) 3mm/0mm, field of view (FOV) 16cm, frequency/phase matrix (Freq/Phase) 320/320, number of excitations (NEX) 2).

Series 2: Coronal Obl T1 with Fat saturation (FS) (TE/TR min full/480, ETL 4, BW 35.71, ST/SG 3.5mm/0mm, FOV 16cm, Freq/Phase 288/288, NEX 3).

Series 3: Coronal Obl Proton Density (PD) FS Forced Recovery Fast Spin Echo (FRFSE XL), TE/TR 40/3660, ETL 10, BW 31.25, ST/SG 3.5mm/0mm, FOV 16cm, Freq/Phase 320/320, NEX 2).

Series 4: Sagittal T2 Obl FRFSE XL (TE/TR 65/3060, ETL 16, BW 41.67, ST/SG 3mm/0mm, FOV 14cm, Freq/Phase 320/320, NEX 2). The GE 8-channel high definition shoulder coil was used for all sequences.

For the abduction-external rotation (ABER) sequence the arm was raised so the palmar aspect of the hand was resting under the patients occiput and their elbow as close to the Table surface as possible. Series 5: Coronal T1 FS Obl ABER (TE/TR min full/480, ETL 4, BW 31.25, ST/SG 3.5mm/0mm, FOV 18cm, Freq/Phase 256/256, NEX 2). The GE 6 channel flex phased array coil was used for the ABER sequence.
