# Supplementary material for: A prospective study of shoulder pain in primary care: Prevalence of imaged pathology and response to guided diagnostic blocks
Source: BMC Musculoskelet Disord. 2011 May 28;12:119. doi: 10.1186/1471-2474-12-119 (PMC3127806; doi:10.1186/1471-2474-12-119)
Supplement: Additional file 6 — Association between x-ray and ultrasound variables and positive anaesthetic responses to subacromial bursa and acromioclavicular joint diagnostic blocks. Table showing additional results for x-ray and ultrasound imaging variables that were not associated with positive anaesthetic responses to subacromial bursa and acromioclavicular diagnostic blocks. [file 1471-2474-12-119-S6.PDF]

Additional file_6: Association between x-ray and ultrasound variables and positive anaesthetic responses to SAB and ACJ diagnostic blocks.

	SAB injection n=196 (PAR n=66)				ACJ injection n=153 (PAR n= 22)			
**Pathology identified on imaging**	% with pathology present reporting PAR	% with pathology absent reporting PAR	OR (95% CI)	Fishers test (*p*-value)	% with pathology present reporting PAR	% with pathology absent reporting PAR	OR (95% CI)	Fishers test (*p*-value)
**X-Ray**								
ACJ pathology	25	35	0.62 (0.26,1.47)	0.312	14	16	2.11 (0.69, 6.52)	0.189
arthropathy/degenerative changes	25	35	0.63 (0.24,1.68)	0.489	17	16	1.86 (0.55, 6.27)	0.296
osteolysis	14	34	0.32 (0.04, 2.74)	0.428	0	17	1.20 (0.13, 10.79)	1.000
Acromion type								
type I	37	31	1.28 (0.71, 2.34)	0.446	19	14	1.69 (0.68, 4.19)	0.352
type II	28	39	0.63 (0.35, 1.15)	0.132	15	18	0.59 (0.28, 1.49)	0.357
type III	75	33	6.24 (0.64, 61.23)	0.109	0	16	2.02 (0.20, 20.30)	0.469
type IV	75	33	0.66 (0.62, 0.73)	1.000	0	15	0.86 (0.80, 0.91)	1.000
os acromiale	75	33	6.14 (0.63, 60.25)	0.112	0	16	0.85 (0.80, 0.91)	1.000
Glenohumeral joint pathology	20	34	0.48 (0.10, 2.31)	0.500	0	17	0.85 (0.80, 0.91)	0.594
degenerative changes	13	35	0.27 (0.03, 2.25)	0.271	0	17	0.85 (0.80, 0.91)	0.594
Rotator cuff calcification	44	32	1.66 (0.71, 3.89)	0.262	13	16	0.30 (0.04, 2.36)	0.312
supraspinatus	56*	31	2.82 (1.00, 7.97)	0.054	25	16	0.58 (0.07, 4.74)	1.000
infraspinatus	29	34	0.79 (0.15, 4.21)	1.000	0	16	0.85 (0.80, 0.91)	0.594
subscapularis	0	34	0.66 (0.59, 0.73)	0.181	0	17	0.85 (0.80, 0.91)	0.594
**Ultrasound**								

ACJ pathology	38	32	1.29 (0.66, 2.53)	0.489	11	16*	1.16 (0.22, 6.17)	0.053
Glenohumeral joint effusion	14	35	0.31 (0.04, 2.65)	0.426	20	15	0.85 (0.80, 0.91)	0.593
Rotator cuff - any pathology	38	30	1.44 (0.79, 2.62)	0.290	13	19	0.48 (0.19, 1.23)	0.166
any tear	42	31	1.60 (0.83, 3.06)	0.175	0	21*	0.32 (0.09, 1.15)	0.082
calcification	35	33	1.11 (0.56, 2.20)	0.861	18	16	0.72 (0.23, 2.28)	0.785
tendinosis	31	34	0.87 (0.37, 2.03)	0.834	25	14	0.59 (0.13, 2.73)	0.740
Supraspinatus pathology	39	30	1.50 (0.83, 2.72)	0.223	12	20	0.72 (0.28, 1.83)	0.643
calcification	49*	31	2.13 (1.00, 4.55)	0.068	22	15	1.40 (0.42, 4.60)	0.526
tendinosis	33	34	0.98 (0.42, 2.33)	1.000	27	14	0.63 (0.14, 2.92)	0.740
tear	40	32	1.43 (0.72, 2.85)	0.369	0	21	0.43 (0.12, 1.55)	0.286
intrasubstance	39	33	1.31 (0.53, 3.20)	0.640	0	18	0.32 (0.04, 2.53)	0.473
partial thickness (BS)	0	34	0 66 (0.59, 0.73)	0.302	0	17	0.85 (0.80, 0.91)	1.000
partial thickness (AS)	25	34	0.65 (0.13, 3.30)	0.720	0	17	2.08 (0.39, 11.05)	0.323
full thickness	70**	32	5.02 (1.25, 20.11)	0.033	0	16	0.84 (0.79, 0.91)	0.630
Infraspinatus pathology	33	34	0.98 (0.29, 3.40)	1.000	0	17	0.84 (0.79, 0.91)	0.217
calcification	33	34	0.98 (0.24, 4.07)	1.000	0	17	0.84 (0.79, 0.91)	0.359
tendinosis	0	34	0.66 (0.60, 0.73)	1.000	0	16	0.86 (0.80, 0.91)	1.000
tear	33	34	0.99 (0.09, 11.06)	1.000	0	16	0.85 (0.80, 0.91)	1.000
intrasubstance tear	100	33	0.33 (0.27, 0.41)	0.337	0	16	0.86 (0.80, 0.91)	1.000
partial thickness	0	34	0.66 (0.60, 0.73)	1.000	0	16	0.86 (0.80, 0.91)	1.000
full thickness	0	34	0.66 (0.60, 0.73)	1.000	0	16	0.86 (0.80, 0.91)	1.000
Subscapularis Pathology	28	35	0.72 (0.30, 1.72)	0.528	17	15	0.25 (0.03, 1.96)	0.203
calcification	25	35	0.63 (0.22, 1.81)	0.462	22	15	0.40 (0.05, 3.19)	0.698
tendinosis	0	34	0.66 (0.60, 0.73)	0.302	33	15	0.86 (0.80, 0.91)	1.000
tear	30	34	0.84 (0.21, 3.35)	1.000	0	17	0.85 (0.79, 0.91)	0.359
intrasubstance	40	34	1.32 (0.22, 8.12)	1.000	0	16	0.85 (0.80, 0.91)	1.000
partial thickness	25	34	0.65 (0.07, 6.39)	1.000	0	16	0.85 (0.80, 0.91)	1.000
full thickness	0	34	0.66 (0.60, 0.73)	1.000	0	16	0.85 (0.80, 0.91)	1.000
Long head of biceps tendon								

tendon sheath effusion	39	33	1.27 (0.54, 2.98)	0.657	46	10	0.59 (0.13, 2.73)	0.740
tendinosis	33	34	0.99 (0.09, 11.06)	1.000	0	16	3.07 (0.27, 35.39)	0.374
tear or rupture	33	34	0.99 (0.09, 11.06)	1.000	0	16	0.85 (0.80, 0.91))	1.000
subluxation	33	34	0.99 (0.09, 11.06)	1.000	0	16	0.85 (0.80, 0.91))	1.000
Subacromial bursa pathology								
bursal fluid/effusion	30	34	0.83 (0.30, 2.27)	0.807	0	18	0.84 (0.78, 0.90)	0.130
calcification	33	34	0.99 (0.09, 11.1)	1.000	0	15	1.17 (1.09, 1.25)	1.000
bursal dimension								
<1.0mm	34	33	1.01 (0.55, 1.87)	1.000	21	13	1.49 (0.60, 3.71)	0.480
≥1mm	33	34	0.99 (0.54, 1.84)	1.000	13	21	0.67 (0.27, 1.67)	0.480
≥2mm	36	33	1.16 (0.58, 2.30)	0.725	16	16	0.84 (0.29, 2.45)	1.000
≥3mm	40	34	1.32 (0.22, 8.12)	1.000	0	16	0.85 (0.80, 0.91)	1.000
bunching								
acromion	37	33	1.18 (0.64, 2.15)	0.645	20	12	0.71 (0.28, 1.81)	0.495
symptomatic bunching (acromion)	39	33	1.26 (0.68, 2.33)	0.528	19	13	0.57 (0.21, 1.56)	0.342
CAL	28	29	0.98 (0.38, 2.35)	1.000	5	14	1.21 (0.29, 4.92)	1.000
symptomatic bunching (CAL)	33	25	1.44 (0.58, 3.60)	0.488	7	12	1.25 (0.31, 5.10)	1.000

Abbreviations: SAB, subacromial bursa; ACJ, acromioclavicular joint; PAR, positive anaesthetic response (≥80% post-injection pain intensity reduction); OR, unadjusted odds ratio for PAR; CI, confidence interval; BS, bursal surface; AS, articular surface; CAL, coracoacromial ligament.

Percentages do not total 100% as these represent proportion of subjects with or without pathology on imaging (row percentages in contingency table) who experienced PAR. Negative anaesthetic response group results are not presented.

† no cases in which pathology was identified and OR could not be calculated.
**significant at p<0.05
*significant at p<0.10
