# Supplementary material for: A prospective study of shoulder pain in primary care: Prevalence of imaged pathology and response to guided diagnostic blocks
Source: BMC Musculoskelet Disord. 2011 May 28;12:119. doi: 10.1186/1471-2474-12-119 (PMC3127806; doi:10.1186/1471-2474-12-119)
Supplement: Additional file 7 — Association between x-ray and ultrasound variables and positive anaesthetic responses to glenohumeral joint diagnostic block. Table showing additional results for x-ray and ultrasound imaging variables that were not associated with positive anaesthetic response to glenohumeral joint diagnostic block. [file 1471-2474-12-119-S7.PDF]

Additional file_7: Association between x-ray and ultrasound variables and positive anaesthetic responses to GHJ diagnostic block.

Pathology identified on imaging	GHJ injection n=73 (PAR n=12) % with pathology present reporting PAR	% with pathology absent reporting PAR	OR (95% CI)	Fishers test (*p*-value)
**X-Ray (n=203)**				
ACJ pathology	14	16	0.85 (0.16, 4.23)	1.000
arthropathy/degenerative changes	17	16	1.06 (0.19, 5.38)	1.000
osteolysis	0	17	0.83 (0.75, 0.92)	1.000
Acromion type				
type I	19	14	1.54 (0.45, 5.34)	0.534
type II	15	18	0.80 (0.23, 2.74)	0.759
type III	0	16	0.84 (0.76, 0.93)	1.000
type IV	0		0.84 (0.76, 0.93)	1.000
os acromiale	0	16	0.84 (0.76, 0.93)	1.000
Glenohumeral joint pathology	0	17	0.83 (0.75, 0.92)	1.000
degenerative changes	0	17	0.83 (0.75, 0.92)	1.000
Rotator cuff calcification	13	16	0.73 (0.08, 6.29)	1.000
supraspinatus	25	16	1.82 (0.17, 18.49)	0.510
infraspinatus	0	16	0.84 (0.76, 0.93)	1.000
subscapularis	0	17	0.83 (0.75, 0.92)	1.000
**Ultrasound (n=203)**				
ACJ pathology	11	16	0.60 (0.12, 3.07)	0.718
Glenohumeral joint effusion	20	15	1.45 (0.15, 14.34)	0.569
Rotator cuff - any pathology	13	19	0.61 (0.18, 2.13)	0.533
any tear	0	21**	0.79 (0.69, 0.90)	0.029
calcification	18	16	1.17 (0.28, 4.90)	1.000
tendinosis	25	14	2.0 (0.45, 8.83)	0.394
Supraspinatus pathology	12	20	0.55 (0.15, 2.03)	0.528
calcification	22	15	1.60 (0.29, 8.84)	0.611
tendinosis	27	14	2.29 (0.51, 10.30)	0.374
tear	0	21**	0.79 (0.69, 0.90)	0.059
intrasubstance	0	18	0.82 (0.73, 0.92)	0.338
partial thickness (BS)	0	17	0.83 (0.75, 0.92)	1.000
partial thickness (AS)	0	17	0.83 (0.75, 0.92)	0.583
full thickness	0	16	0.84 (0.76, 0.93)	1.000
Infraspinatus pathology	0	17	0.83 (0.75, 0.92)	1.000
calcification	0	17	0.83 (0.75, 0.92)	1.000
tendinosis	0	16	†	†
tear	0	16	†	†
intrasubstance tear	0	16	†	†
partial thickness	0	16	†	†
full thickness	0	16	†	†

Subscapularis Pathology	17	15	1.06 (0.20, 5.59)	1.000
calcification	22	15	1.60 (0.29, 8.84)	0.636
tendinosis	33	15	2.77 (0.23, 33.27)	0.421
tear	0	17	0.83 (0.75, 0.92)	1.000
intrasubstance	0	16	0.84 (0.76, 0.93)	1.000
partial thickness	0	16	0.84 (0.76, 0.93)	1.000
full thickness	0	16	0.84 (0.76, 0.93)	1.000
Long head of biceps tendon				
tendon sheath effusion	46**	10	8.00 (2.02, 31.72)	0.004
tendinosis	0	16	0.84 (0.76, 0.93)	1.000
tear or rupture	0	16	0.84 (0.76, 0.93)	1.000
subluxation	0	16	0.84 (0.76, 0.93)	1.000
Subacromial bursa pathology				
bursal fluid/effusion	0	18	0.82 (0.74, 0.92)	0.589
calcification	0	16	†	†
bursal dimension				
<1.0mm	21	13	1.86 (0.54, 6.47)	0.519
≥1mm	13	21	0.54 (0.16, 1.86)	0.524
≥2mm	16	16	0.90 (0.22, 3.73)	1.000
≥3mm	0	16	†	†
bunching				
acromion	20	12	1.85 (0.51, 6.74)	0.328
symptomatic bunching (acromion)	19	13	1.55 (0.42, 5.68)	0.496
CAL	5	14	0.35 (0.03, 3.70)	0.610
symptomatic bunching (CAL)	7	12	0.52 (0.05, 5.55)	1.000

Abbreviations: GHJ, glenohumeral joint; PAR, positive anaesthetic response (≥80% post-injection pain intensity reduction); CAL, coracoacromial ligament; OR, unadjusted odds ratio for PAR; CI, confidence interval; BS, bursal surface; AS, articular surface.

Percentages do not total 100% as these represent proportion of subjects with or without pathology on imaging (row percentages in contingency table) who experienced PAR. Negative anaesthetic response group results are not presented.

† no cases in which pathology was identified and odds ratios could not be calculated.

**significant at $p<0.05$

*significant at $p<0.10$
