# Supplementary material for: A prospective study of shoulder pain in primary care: Prevalence of imaged pathology and response to guided diagnostic blocks
Source: BMC Musculoskelet Disord. 2011 May 28;12:119. doi: 10.1186/1471-2474-12-119 (PMC3127806; doi:10.1186/1471-2474-12-119)
Supplement: Additional file 8 — Association between magnetic resonance arthrogram variables and positive anaesthetic responses to glenohumeral joint diagnostic block. Table showing additional results for magnetic resonance imaging variables that were not associated with positive anaesthetic response to glenohumeral diagnostic block. [file 1471-2474-12-119-S8.PDF]

Additional file_8: Relationship between MRA findings and positive anaesthetic responses to glenohumeral joint diagnostic block.

Pathology identified on MRA	GHJ injection n=73 (PAR n=12) % with PAR when pathology present	% with PAR when pathology absent	OR (95% CI)	Fishers test (*p*-value)
**Acromioclaviclular joint pathology**	20	11	2.03 (0.50, 8.23)	0.516
arthropathy/degenerative changes	14	19	0.72 (0.21, 2.49)	0.751
osteolysis	40	15	3.93 (0.58, 26.58)	0.187
os acromiale				
**Glenohumeral joint pathology**	14	21	0.59 (0.17, 2.05)	0.524
degenerative changes	0	18	†	0.591
labral tear	12	21	0.53 (0.15, 1.96)	0.360
paralabral cyst	22	16	1.57 (0.28, 8.69)	0.636
rotator interval pathology	15	21	0.59 (0.17, 2.06)	0.574
synovitis- inferior recess	22	16	1.54 (0.28, 8,53)	0.639
synovitis– rotator interval	14	18	0.69 (0.19, 2.55)	0.752
**Rotator cuff - any pathology**	11	27	0.34 (0.10, 1.21)	0.101
any tear	9	23	0.33 (0.08, 1.35)	0.124
tendinosis	9	20	0.42 (0.08, 2.10)	0.326
Suprapinatus pathology	12	22	0.52 (0.15, 1.81)	0.345
tendinosis	11	18	0.58 (0.11, 2.91)	0.718
tear	11	20	0.49 (0.12, 2.00)	0.350
intrasubstance	0	19	0.81 (0.72, 0.91)	0.198
partial thickness – bursal surface	0	18	0.83 (0.74, 0.92)	0.583
partial thickness – articular surface	22	16	1.57 (0.28, 8.69)	0.636
full thickness	25	16	1.79 (0.17, 18.80)	0.521
Infraspinatus pathology	0	19	0.82 (0.72, 0.91)	0.198
tendinosis	0	17	0.83 (0.75, 0.92)	1.000
tear	0	18	0.82 (0.74, 0.92)	0.581
intrasubstance tear	0	17	0.83 (0.75, 0.92)	1.000
partial thickness	0	17	0.83 (0.75, 0.92)	1.000
full thickness		16	†	†
Subscapularis Pathology	0	19	0.82 (0.73, 0.92)	0.339
tendinosis	0	17	0.83 (0.75, 0.92)	1.000
tear	0	18	0.83 (0.74, 0.92)	0.583
intrasubstance	0	17	0.83 (0.75, 0.92)	1.000
partial thickness		16	†	†
full thickness	0	17	0.84 (0.76, 0.93)	1.000
**Long head of biceps tendon**				0.583
tendinosis	0	17	0.83 (0.75, 0.92)	1.000
tear or rupture	0	17	0.83 (0.75, 0.92)	1.000
**Subacromial bursa**				0.725
bursal thickening	20	15	1.47 (0.27, 8.08)	0.648
bursitis	15	20	0.71 (0.19, 2.69)	0.725
contrast seen in bursa on MRI	33	15	2.90 (0.47, 17.99)	0.254

GHJ, glenohumeral joint; PAR, positive anaesthetic response (≥80% post-injection pain intensity reduction); MRA, magnetic resonance arthrogram; MRI, magnetic resonance imaging.

† no cases in which pathology was identified- association could not be calculated.
**significant at $p<0.05$
*significant at $p<0.10$
